# Chemical Characterization and Sensory Evaluation of Scottish Malt Spirit Aged in Sherry Casks^®^: Comparison Between Static and Dynamic Aging Systems

**DOI:** 10.3390/molecules30061378

**Published:** 2025-03-19

**Authors:** Daniel Butrón-Benítez, Manuel J. Valcárcel-Muñoz, M. Valme García-Moreno, M. Carmen Rodríguez-Dodero, Dominico A. Guillén-Sánchez

**Affiliations:** 1 Departamento de Química Analítica, Facultad de Ciencias, Instituto Investigación Vitivinícola y Agroalimentaria (IVAGRO), Universidad de Cádiz, Campus Universitario de Puerto Real, 11510 Puerto Real, Cádiz, Spain; daniel.butron@uca.es (D.B.-B.); maricarmen.dodero@uca.es (M.C.R.-D.); 2 Bodegas Fundador S.L.U., C/San Ildefonso, nº 3, 11403 Jerez de la Frontera, Cádiz, Spain; 3 Oeno R&D FL, 11402 Jerez de la Frontera, Cádiz, Spain; mjc.valcarcel@gmail.com

**Keywords:** whisky, aging, static system, dynamic system, average age, malt, Sherry Cask, partial least squares, multiple linear regression

## Abstract

Aging spirits in wooden casks is a traditional and mandatory process for the production of certain products, such as whisky. The physicochemical and sensory changes that occur during aging are shaped by the characteristics of the barrels and the aging method used. In this paper, we examined the behavior of the same malt spirit when aged using two different Sherry Casks^®^ methods. The first one was static aging, with the distillate remaining still in the cask, and the second one was a dynamic system, characterized by the regular racking of the spirit between casks at different aging stages (*Criaderas and Solera*). For 36 months, the aging spirits were sampled and analyzed to determine any changes in acidity, volatile, and phenolic compound content that might indicate changes in their chemical profile. The spirits were also subjected to sensory evaluations. The analysis revealed a significant evolution of the distillate in either system, although with different chemical profiles. Multiple Linear Regression Models (MLR and PLS) were successfully used to estimate the age of the distillates at a high level of confidence. Although, after the first racking operation, the distillates in the dynamic system had an average age greater than the theoretical one, these differences tended to fade away as the system gradually stabilized.

## 1. Introduction

The aging of spirits, such as whisky, brandy, rum, grappa, etc., in wooden casks is a traditional process that has been demonstrated to improve the organoleptic qualities of the final products, which acquire a complex and unique character through aging [1,2]. Global production of spirituous beverages reached 33,800 million liters in 2023, with aged distillates holding high relevance in the sector as they are highly valued by consumers [3]. Such is the importance of the aging process, which has become a mandatory requirement for the production of whisky [4].

Whisky is a spirit produced exclusively via distillation at less than 94.8% alcohol by volume (ABV) of a malted cereal mash, whether or not the mash bill contains or not whole grains of unmalted cereals, and which is then saccharified and fermented by the action of yeast. The distillates obtained must exhibit the aroma and flavor derived from the raw materials used and they must have been aged for at least three years in wooden casks of 700 L volume or less [4].

The physicochemical and organoleptic changes that take place during the aging of the distillates, and that have an important role with regard to the quality of the final product, are conditioned by a number of variables, among which the type of cask used and the aging method are of particular significance [5,6,7]. The casks, which are generally oak-wood made, are characterized by their specific manufacturing process and by their previous usage. When casks that have previously contained Sherry wine, i.e., Sherry Casks^®^, are used, they confer unique attributes to the aged distillates [8,9]. Some whisky producers in Scotland, Japan, Spain, etc., use them for the last years of aging, which implies that their whiskeys spend an important part of their aging time either in new oak casks or in casks that had previously contained bourbon. Then, they are transferred to Sherry Casks^®^ to complete their aging process and acquire their characteristic profile and a more complex organoleptic character [2,10,11,12].

As for the aging method in casks, two different approaches have been traditionally employed: static aging, which involves having the distillate in the casks for a specific period of time until it is eventually bottled; and dynamic aging (also known as *Criaderas and Solera*), which employs a set of casks arranged at different levels of aging, and where only the distillate from the most aged level is partially extracted and bottled. As the oldest spirit is extracted, it is replaced with distillate from the next oldest cask level (first Criadera), and so on until the distillate from the youngest level (last Criadera) is refilled with new unaged distillate [4].

This periodic transference of the distillates promotes continuous blending and more uniform aging over the years. The transferring and blending of the distillates also favor their aeration and, consequently, enhance the oxidation of some of their compounds, which in turn has an impact on the flavor and taste of the final product [7,13,14]. Static aging has been traditionally used in the spirits industry for the production of whisky, rum, and brandies, such as Armagnac and Cognac, while dynamic aging has been used by some rum producers and is particularly characteristic of Sherry Brandy making.

The objective of this work is to characterize the physicochemical and organoleptic features of a malt spirit aged both statically and dynamically, in order to compare whisky obtained through the traditional static aging method against that obtained through a dynamic system. This research should be an important contribution to the aged, distilled beverages sector, as it provides interesting insights into the evolution of a malt spirit when aged using two different industrial-scale aging systems.

The malt spirits were evaluated through the physicochemical and sensory analyses of samples aged for different lengths of time over the 3 years of the experiments. In order to compare the two aging systems, Multiple Linear Regression (MLR) and Partial Least Squares (PLS) models were used in order to correlate the physicochemical parameters analyzed with the actual age of the whiskies. Both models allowed us to corroborate a close correlation between the parameters analyzed and the aging time of the whiskies. It has been concluded that chemometric techniques are a tool of great potential and interest for the industry to develop a deeper understanding of the aging process and its impact on the final product, as well as an effective way to determine which variables and parameters should be taken into consideration for more efficient and accurate control of the aging process.

## 2. Results and Discussion

The discussion of the results has been focused on the comparison between the characteristics of the same malt spirit aged either in a static system or in a dynamic system for 3 years, for which exclusively Sherry Casks^®^ of the same characteristics have been used. Therefore, in order to uncover the differences between static and dynamic aging, only the results corresponding to the samples from the Solera level (the SRA samples), i.e., the final product of the dynamic system, have been considered.

### 2.1. Alcoholic Strength

**Figure 1:**
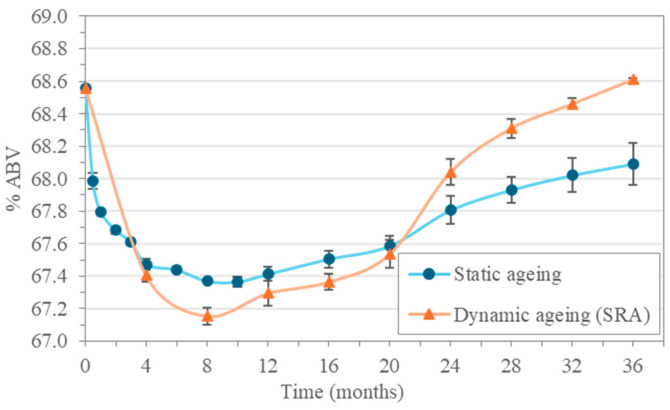
Evolution of alcoholic strength in the static aging malt spirits and in the Solera (SRA) of the dynamic system.

The evolution of the alcoholic strength of whisky or malt spirit aged for 36 months can be seen in Figure 1.

During the first months of static aging, the malt spirit experiences a decrease in its alcohol content as a result of both its dilution with the Oloroso Sherry wine that had been previously contained in the casks and also because of the evaporation of alcohol during the cask-filling operation. This alcohol reduction turns more gradual as the process continues and then it reaches a period with little changes at approximately 8–10 months of aging. From that moment on, a steady increase in alcohol content follows, as water content decreases because of the transpiration and evaporation that takes place through the casks’ pores—an effect known as “*merma*” [15]. All in all, an increase of approximately 0.70% ABV was observed to take place over the 12–36 month period.

A similar effect was observed in the Solera (SRA) malt spirits, which initially showed a greater decrease in alcohol content due to the dilution of the Oloroso Sherry wine from the casks and the loss of alcohol by evaporation during the filling of the Criadera and Solera cask arrangement. Such losses built up as a result of the first two replenishment operations. However, as more extraction and replenishment operations were carried out, there was a gradual increase in the alcohol content, which, after 36 months, reached that of the initial distillate. This evolution is explained by the way the dynamic system of *Criaderas and Solera* works, as the aged spirit is extracted from the Solera and replaced with the spirit, of a higher alcoholic strength, from the Criadera (CRA), which has been gradually recovering its alcoholic strength as the extracted portion were replaced with unaged malt spirit.

### 2.2. pH, Total Acidity, Volatile Acids, and Organic Acids

The pH values are shown in Table S1 in the Supplementary Materials. There, it can be seen that there was an initial decrease in the pH of the spirit as the casks were filled, falling from 5.21 for the unaged malt spirit to 4.57 for the 0.5-month-old sample. From that point on, the pH remained stable, although significant changes were registered over the 36 months of aging either in a dynamic or static system. The static system showed a slight decrease in pH, while the dynamic system showed an increase from a sample of 4 months to 36 months.

**Figure 2:**
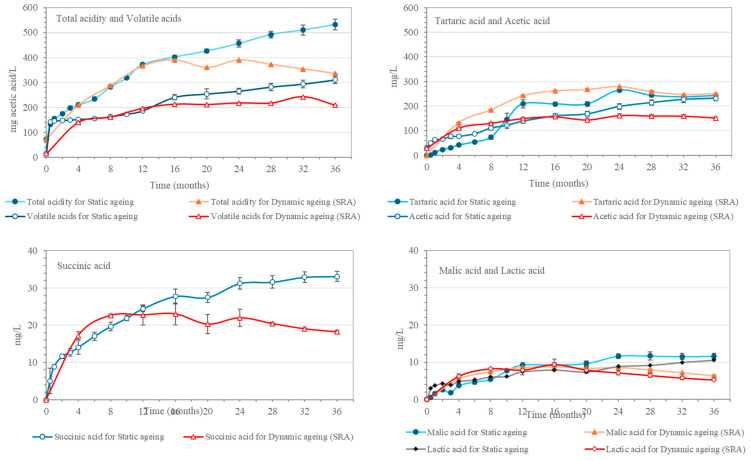
Evolution in the static aging malt spirits and in the Solera (SRA) of the dynamic aging system: total acidity and volatile acids, tartaric acid and acetic acid, succinic acid, and malic and lactic acids.

These results are in agreement with those reported for total acidity and volatile acids, which, after an initial rapid increase after 0.5 months, registered a continuous increment over the rest of the 36 months (Figure 2). This increase was caused by the higher concentration of organic acids that occurs during the aging process, mainly due to the contributions from the Sherry Casks^®^ seasoning wine [9]. Thus, tartaric, malic, and succinic acids all showed similar trends and increased during the first 24 months. Then, as they reached their highest concentrations, their evolution tended toward stability. With regard to acetic acid, the wood and the oxidation as well as the “*merma*” played significant roles [5,16,17]

The total acidity and volatile acids in the spirit aging in the dynamic system increased by similar values as those corresponding to the spirit in the static aging system, although such increments were less pronounced after 16–20 months of aging and total acidity even showed a drop after 24 months of aging. This decrease in total acidity coincided in time with the sixth extraction and replenishment operation, which reveals that, at this stage, the casks’ wood was yielding a lower amount of acids than that contained in the aged spirit samples extracted from the Solera.

### 2.3. Aldehydes, Methanol, and Alcohols

The concentration of acetaldehyde depends on the distillation, and its equilibrium with its acetal formation depends on the alcoholic strength and the pH of the distillate. The evolution of total acetaldehyde concentration in malt spirits during aging is also influenced by processes of evaporation and oxidation into acetic acid and formation through ethanol oxidation [2]. Thus, an increase in the concentration of total acetaldehydes has been observed over the 36 months of static aging (Table S2).

Benzaldehyde is also present in the distillate, as it is indirectly derived from the degradation of amino acids during the malting of the grain and subsequently from the distillation process [18]. After the initial dilution that has been observed to take place during the cask filling process, only a slight increase was registered over the 36 months of the static process, which may be explained by the concentration that results from the “*merma*” phenomenon.

An increase in the total acetaldehydes in SRA has been observed up to 12–16 months and from then on it starts to fall down to values close to those of the unaged spirit, perhaps due to the fact that the continuous extraction and replenishment operations facilitate the evaporation of these highly volatile compounds, as well as to the aeration involved in the dynamic system, which causes the oxidation of the acetaldehyde into acetic acid (Table S2). Meanwhile, benzaldehyde does not present any evolution throughout the aging process studied.

The methanol level in the distillate is determined by the process of distillation [19] and the slight increase observed in the static aging samples may be a consequence of increases due to “*merma*”, contributions from the Sherry Cask^®^ seasoning wine and/or the lignin, hemicellulose or xylans of the wood [5,16,20]. This slight increase is not observed in the evolution of the SRA, which presents similar methanol levels at the start and after 36 months of aging [5,16,20].

Higher alcohols (HA) are formed during alcoholic fermentation and have a major role in the aroma either by themselves or as precursors of other compounds that are also involved in the aroma of the distillates [18]. The amount of HA in the distillate is marked by the characteristics of the distillation process [18,19,21]. The results show how in the static aging system HAs remain constant for up to 10–12 months, after which they begin to increase slightly until 36 months of aging, probably due to the higher concentration resulting from “*merma*”, as this coincides with the evolution of alcoholic strength (Tables S3 and S4). In the case of the SRA, the same initial decrease is observed as in the static system, but after 12 months, which corresponds to the third extraction and replenishment operation, and until the end of the aging process, no significant changes were observed. These results suggest that the increases in the concentrations of these alcohols due to the effect of “*merma*” are similar to those caused by the removal of these compounds during the periodic extractions of liquid from the Solera. Thus, the dynamic system keeps an equilibrium of higher alcohol through the extractions and replenishments.

**Figure 3:**
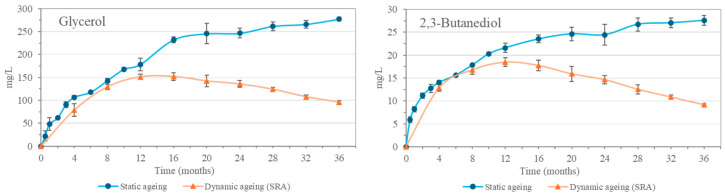
Evolution of glycerol and 2,3-butanediol in the statically aged malt spirits and in the Solera (SRA) from the dynamic aging system.

Polyalcohols characteristic of aging in Sherry Cask^®^, such as glycerol and 2,3-butanediol, were also found in the malt spirits analyzed. These compounds were not initially present in the distillate but increased in concentration with aging (Figure 3), in accordance with previous works [9]. They reached relatively high concentrations, 277.5 mg/L glycerol and 27.6 mg/L 2,3-butanediol in the static aging system. While the SRA reached a maximum at 12–16 months with 151.3 mg/L of glycerol and 18.5 mg/L of 2,3-butanediol, after which a decrease was observed until the 36th month of aging. This trend, which indicates a lesser contribution of these compounds from the casks in the dynamic system compared to the initial one, is also explained by the extraction and replenishment operations, where the extraction of the aged spirit from the Solera, and its replacement with a liquid with a lower concentration of these compounds, such as the Criadera (CRA), results in a progressive dilution. The CRA has a smaller amount of these polyalcohols, which are obtained during aging as the replenishments are carried out using the unaged malt spirit, which does not contain these compounds, resulting in greater dilution.

### 2.4. Esters

Esters are among the most significant groups of congeners in the distillate and are key to the flavor and aroma of the final whisky. They include ethyl acetate and fatty acid ethyl esters (FAEE). Other ethyl esters are obtained exclusively through aging, such as the ethyl esters of organic acids: diethyl succinate, diethyl tartrate, and diethyl malate.

**Figure 4:**
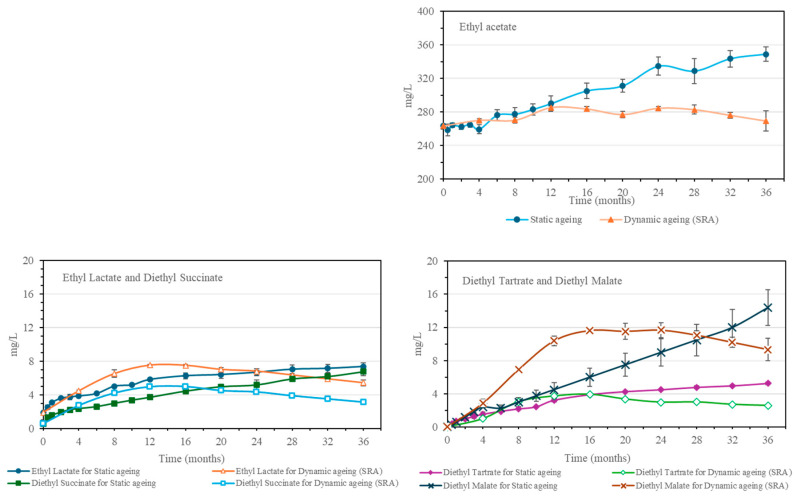
Evolution of ethyl acetate, ethyl lactate and diethyl succinate, and diethyl tartrate and diethyl malate, in the static aging system and in the Solera (SRA).

Ethyl acetate, the most abundant ester, is formed by the esterification of acetic acid and ethanol. It is present in the unaged distillate, and it originated both during the alcoholic fermentation and during the distillation [19,21]. It has been observed that the concentration of ethyl acetate increases during the aging process (Figure 4) as a result of the esterification of the acetic acid present in the distillate and the ethanol [2,18]. Its increase in the static system has been observed to take place over the whole aging time, while in the dynamic system, it only increases for the first 12 months and then remains constant.

FAEEs are a group of volatile compounds of major importance in the composition of whisky, as they are among the main compounds responsible for its aroma. They are formed during alcoholic fermentation and the conditions of both fermentation and distillation play a major role in the amount of esters found in the final distillate [18,19,22]. During aging, the concentration of FAEE increases due to the esterification of the free fatty acids that are present in the distillate and/or are concentrated by “*merma*” [2,15]. In the static system, a gradual increase has been observed, reaching 173.3 mg/L at 36 months (Table S5). The SRA malt spirits, on the other hand, increased up to 181 mg/L after 12 months of aging, and from there on, they did not present any significant evolution until the end of the aging period (Table S6).

The organic acid esters identified are diethyl succinate, diethyl tartrate, and diethyl malate, which are derived from the esterification of ethanol with succinic acid, tartaric acid, and malic acid, respectively. These three esters, similar to what has been observed with the acids, are not present in the distillate (Figure 4). They are obtained directly and indirectly through the aging of the distillate in the Sherry Casks^®^ as they are a direct contribution of the esters from the cask-seasoning wine or indirectly formed through the esterification of the acids yielded by the casks during the spirit aging process. The trends observed were similar to those of other compounds obtained during aging. In the static aging system, an increase in all the organic acid esters studied was observed throughout the 36 months. As for SRA, a maximum value was reached between the 12th and the 16th month, at which point a slight decrease was observed, which was more pronounced for ethyl lactate and diethyl succinate and more gradual for diethyl tartrate and diethyl malate.

### 2.5. Total Phenolic Index, Phenolic Compounds, and Furanic Aldehydes

Malt spirit increases its Total Polyphenol Index (TPI) with cask aging time. This is due to the increasing amount of phenolic compounds and furanic aldehydes that are yielded by the Sherry Casks^®^ wood [23]. These compounds depend on the characteristics of the casks and contribute to the organoleptic qualities of the spirit, providing color, aroma, flavor, and astringency, among others [16]. In the static aging system, the TPI value increases throughout the 36 months, while in the SRA malt spirits, the maximum value was found in the 12–16-month-old samples, after which it remained stable and without any noticeable evolution until the 32–36-month period when the TPI value presents a falling trend.

**Figure 5:**
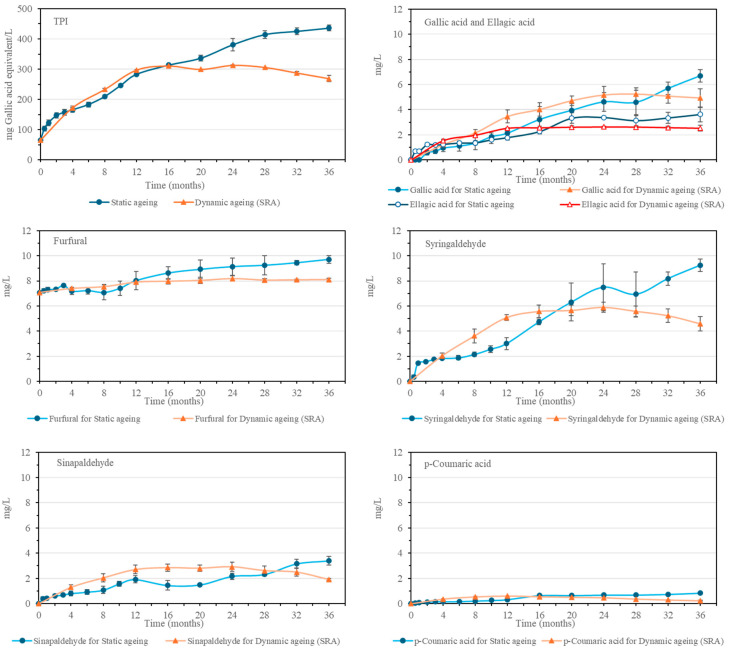
Evolution of Total polyphenol index (TPI), gallic acid and ellagic acid, furfural, syringaldehyde, sinapaldehyde, p-coumaric acid in static aging and in the Solera (SRA) from the dynamic aging system.

Similarly, the phenolic compounds and furanic aldehydes that were identified also presented a tendency to increase during the aging time in wood. Detailed data can be seen in Figure 5 and Figure S1 in the Supplementary Materials. Furfural was the only compound found in the initial distillate, as it derives from the dehydration of the pentoses during the heat treatment [20]. Likewise, its concentration increases during aging as a result of the contributions from the hemicellulose of the wood [16]. In general, the rest of the compounds from this family, i.e., gallic acid, 5-hydroxymethylfurfural, protocatechuic acid, vanillic acid, p-hydroxybenzaldehyde, 5-methylfurfural, syringic acid, vanillin, syringaldehyde, coniferaldehyde, sinapaldehyde, and ellagic acid, cannot be found in the initial distillate, as they come mainly from the wood of the Sherry Casks^®^, although certain compounds, such as gallic acid, caffeic acid or p-coumaric acid among others, come from both the wood and the cask seasoning wine since they can be found in unaged Oloroso Sherry in considerable quantities [16,24,25].

In general, an increase in these compounds has been observed both during static and dynamic aging, especially with regard to syringaldehyde and gallic acid, which are the compounds that increased the most during the 36 months of the process studied. In the static aging system, the increase occurred over the 36 months of aging, although at different rates, since some compounds, such as ellagic acid, p-coumaric acid, protocatechuic acid, and vanillic acid, increased their content more rapidly during the first 16–24 months and then continued to increase, even if more gradually. In the case of the SRA from the dynamic system, the increase in these compounds reached its maximum in the 12-month malt spirit, similar to other previously mentioned compounds obtained through the aging process. It is worth noting the greater initial increase in p-coumaric acid, sinapaldehyde, p-hydroxybenzaldehyde, syringic acid, and 5-methylfurfural, which presented higher concentrations during the first months than those found in the static process. Subsequently, due to the decrease in concentrations caused by the extraction and replenishment operations, the concentrations measured in the 36-month malt spirit became similar or lower than those corresponding to the static aging system malt spirits.

### 2.6. Regression Model to Determine the Aging Stage of the Spirits

#### 2.6.1. Correlation Study

In order to perform the regression model with the data matrix obtained from the static aging samples, a number of variables were previously selected. Fifty variables were analyzed, all of which exhibited more or less pronounced variations throughout the static aging process. In order to identify the physicochemical parameters that might have a correlation with the aging of the distillate, all of the study variables were correlated with the dependent variable (time in months). The results showed the relationships between the variables and their potential impact on the aging of the distillate. The results are shown in Table 1. Variables with a positive correlation with aging time and r values > 0.6 were identified, i.e., those variables that directly increased their concentration with aging time, such as total acidity or related variables, such as volatile acids, tartaric acid, acetic acid, and ethyl acetate among others. Phenolic compounds and furanic aldehydes, with TPI, gallic acid, vanillin, syringaldehyde, and coniferaldehyde, among other compounds obtained directly from the cask wood, also presented a close correlation with the aging time for static aging. On the other hand, low correlations were found for pH, acetaldehyde, most of the higher alcohols, and the medium-chain fatty acid ethyl esters (ethyl hexanoate, ethyl octanoate, and ethyl decanoate). In the case of pH, there was no significant variation with aging time, while higher alcohols and fatty acid ethyl esters, which are compounds found in the initial distillate, only exhibited slight variations with aging time.

#### 2.6.2. Factor Analyses

After the variables had been selected, a factor analysis was performed using the 39 selected variables that had a correlation greater than 0.6. This allowed us to identify the groups of variables that represent influencing factors on the average age. Eigenvalue factor selection criteria greater than 1.0 and varimax rotation were applied. Under these conditions, two factors were obtained that explained 88.5% of data variability.

**Figure 6:**
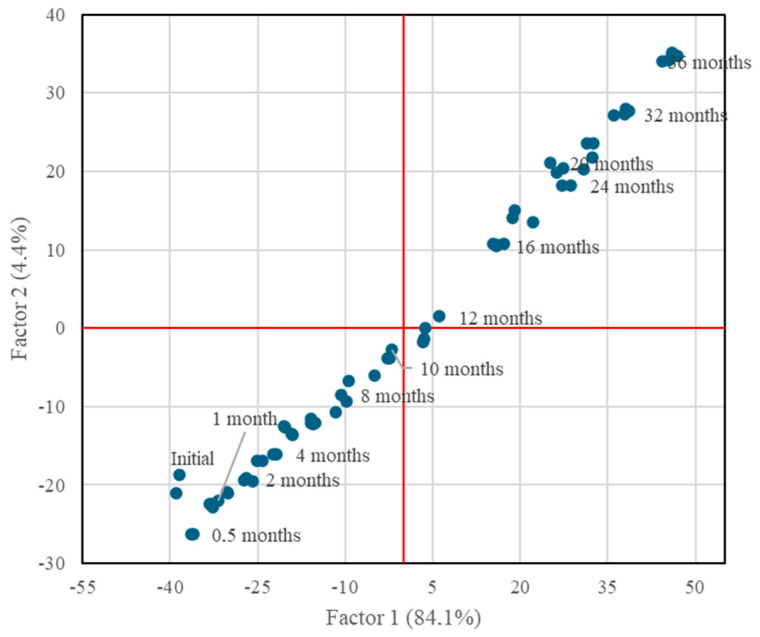
Projection of the statically aged malt spirits on the plane formed by the two factors identified.

The projection of the two factors that had been identified displayed a linear trend, where the statically aged malt spirits were separated and ordered from shorter to longer age time from left to right for Factor 1 and from bottom to top for Factor 2 (Figure 6). So, both factors separated the malt spirits in the same way. The coefficients of the variables for both factors after the most significant varimax rotation (for r >|0.40|) are shown in Table 2.

All the variables that constitute the loading factors of Factor 1, which explains the aging time with 84.1% variability, have a coefficient greater than 0.4, except for ethyl tetradecanoate and benzaldehyde. In other words, all the selected variables have an important weight in explaining Factor 1 and, consequently, the aging time, except for the two variables previously mentioned, which were discarded for the construction of the model.

#### 2.6.3. Regression Model

*Multiple Linear Regression (MLR)*: The statistical analysis was aimed at determining those variables that could have a direct relationship with the aging time of the static aged malt spirit. These variables would be used as markers to estimate the aging time of dynamic aged samples. The statistical technique used for this purpose was Multiple Linear Regression (MLR), for which variables were selected stepwise forward with a *p*-value less than 0.05.

**Figure 7:**
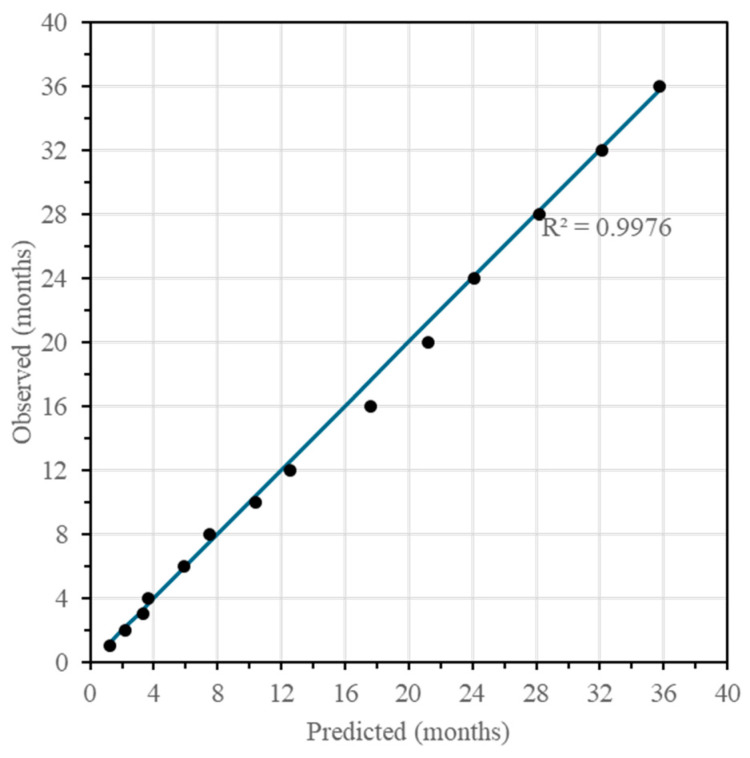
Validation of the model using fourteen samples of aged malt spirit.

Of the 37 variables identified, 9 were selected for the regression model, resulting in a model with a significance level of 95% and an R2 (adjusted to DF) of 99.77% (Table 3). The predicted age and residual of the model is shown in Table S7. The models were performed with 46 cases and 14 were left out so that they could be used to validate the model obtained (Figure 7 and Table S8).

**Figure 8:**
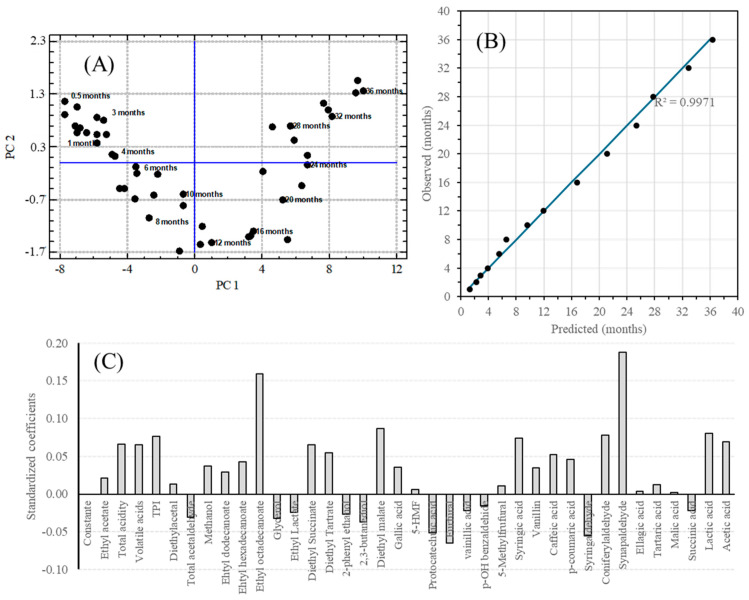
(**A**) Values of PC1 versus the PC2 values obtained from the model based on PLS, (**B**) validation of the model obtained through PLS against the fourteen samples of aged malt spirit, and (**C**) contributions from the individual variables to the principal component in the regression model obtained through PLS.

*Partial Least Square (PLS)*: A PLS regression method was used to construct a predictive model of average aging time as a function of the variables selected for the study. The regression model was performed using 46 cases in static aging between 0.5 and 36 months and was validated using the remaining 14 cases. The results obtained are shown in Figure 8A,B. The regression model obtained for six principal components (PC) presented a regression coefficient of 99.234, with mean squared errors of 5.38, 2.19, 1.51, 1.20, 1.10, and 1.01 for each PC. The predicted age and residual of the model is shown in Table S7. This model predicts the average age with a good fit, as can be seen from the slope of the predicted age and the actual average age close to 1 (0.9941) of the 14 cases used to test the model (Figure 8B and Table S9). Figure 8C and Table S10 show the contribution of each variable to the model obtained.

#### 2.6.4. Determining the Average Age of the Spirits in the Dynamic System

The model was used to predict the average age of the samples extracted every 4 months from the dynamic system over the 36 months of the process studied. Table 4 shows the results obtained from both models and their differences with the actual average age. The predicted average age of the SRA malt spirits exhibited negative age values, i.e., the samples have a higher theoretical age, according to the model, than their actual average age. For SRA-12, the difference between the age predicted by the MLR model and the actual average age increased until 15.5 months of aging, and the same applies to the PLS model until 13.1 months of aging. From that moment on, the samples of subsequent extraction presented lesser differences with respect to the values predicted by the model. Thus, the actual average age was gradually getting closer to that predicted by the model. This evolution is explained by the effect of the extraction and replenishment operations, as the extractions from the Solera are replaced with the liquid extracted from the Criadera, i.e., with an aged spirit of similar composition but at a lower concentration. The dynamic system presents a more noticeable initial evolution than the static one, as the extraction and replenishment operations favor wood extractions and oxidation reactions. Later on, the compounds obtained from the wood gradually deplete and they are, therefore, contributed at a lower rate. This means that the first extractions are logically richer in these compounds, while they are more scarce in the subsequently extracted malt spirits. This, when applied to the predictive model, results in predicted age values gradually closer to the actual average age of the spirits.

### 2.7. Sensory Analysis

Tables S11 and S12 present mean scores that the panel granted to the samples for olfactory and olfactory-gustatory descriptors, respectively, as well as the results of the Analysis of Variance applied to each comparison of samples of similar age according to the different criteria.

No olfactory or olfactory-gustatory defects were detected in any of the samples, so these data have not been included in the tables. The soapy and fatty descriptors also reached very low scores, being absent for most of the judges, and the malted cereal and cooked vegetable odors were also perceived at low intensities. The descriptors with the highest average scores were vinous olfactory note and aromatic intensity, which ranged from medium to high intensities. The remaining descriptors ranged from weak to medium intensities.

The descriptive results have been compared by means of Analysis of Variance according to two criteria, firstly, the average age described in Table 5, and secondly, the total processing time.

**Figure 9:**
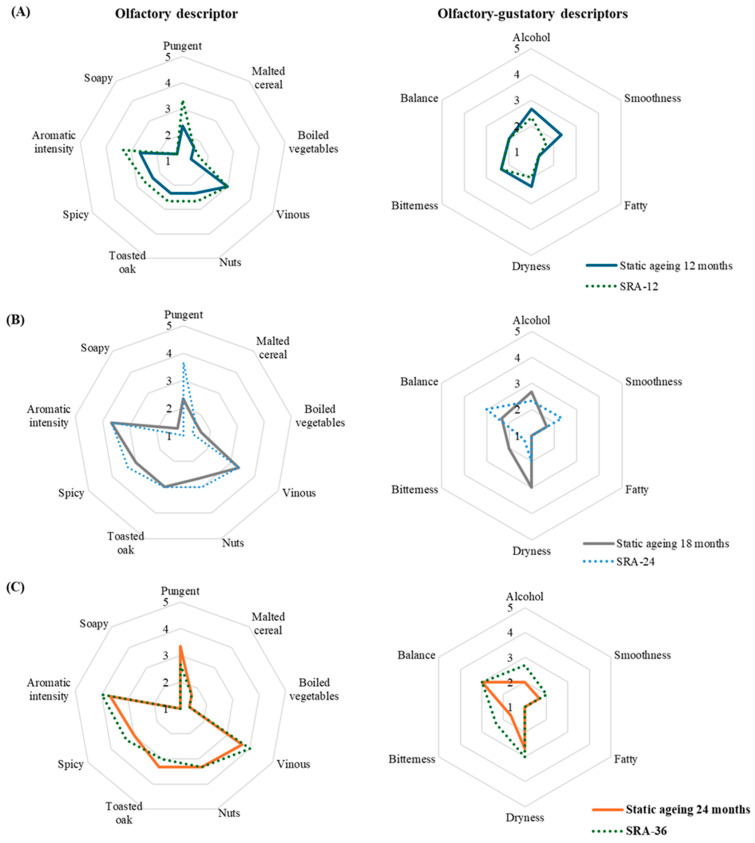
Olfactory and olfactory-gustatory descriptors in the average ages (**A**) 12 months, (**B**) 18 months, and (**C**) 24 months old static and dynamic malt spirits.

The comparison of the samples of malt spirit showed very few significant differences in terms of average age (Figure 9). On the nose, the comparison of samples with an average age close to 12 months (12 months of static aging and SRA-12 (11.7 months average age)) and 18 months (18 months static aging and SRA-24 (18.7 months average age)) showed significant differences and in both cases the pungent sensation of the static aging sample was significantly lower. No difference was confirmed between the static malt spirits approaching 24 months of aging (24-month-old static samples and SRA-36 (22-month average age)).

**Figure 10:**
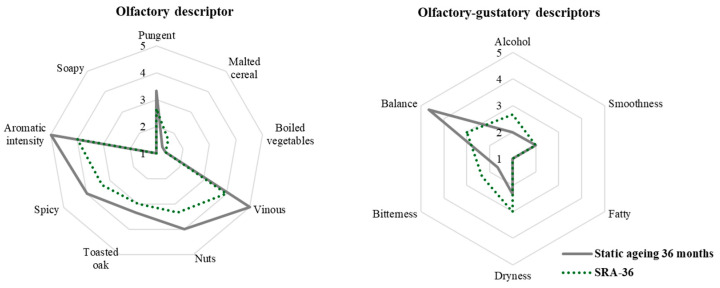
Olfactory and olfactory-gustatory descriptors in the 36-month-old static and dynamic malt spirits.

Certain sensory differences with processing time were statistically confirmed, although this only applied to the oldest samples (36 months) (Figure 10). Thus, the 36-month static malt spirit achieved the highest scores for the olfactory notes of vinous, dried fruit, and aromatic intensity. Mouth balance followed the same pattern, while alcoholic sensation was perceived as significantly lower in the 36-month-old static malt spirit.

In addition, judges were asked to place samples from the Solera (SRA-12, SRA-24, and SRA-36) on a scale with static samples (at 4, 8, 12, 18, 24, and 36 months). Placement on that scale was based on finding the sample from the static system that most closely resembled it. The judges considered the SRA-12 sample to be very similar to the 8-month static sample; the SRA-24 was unanimously placed above the 18-month static sample; and finally, the SRA-36 sample was placed at varying distances from the ends of the 18- and 24-month-old range of the static samples.

## 3. Materials and Methods

### 3.1. Materials

The distillate used for this study was produced in Scotland by the distillation of malted, saccharified, and fermented cereal stocks at 68.8% ABV in copper stills. The 500 L casks used come from the same batch and were made for the same producer from American oak (*Quercus alba*) medium toast wood and seasoned for 3 years with Oloroso Sherry wine at 18% ABV (Sherry Cask^®^) [8]. After the seasoning period, the casks were emptied and allowed to drain for 72 h before being refilled with 485 L of malt spirit (97% cask capacity). The distillate and the Sherry Casks^®^ were supplied by Bodegas Fundador S.L.U., a winery from the “Jerez-Xérès-Sherry” Denomination of Origin. The average humidity and temperature of the cellar remained constant at 71.5 ± 7.7 g/m3 and 19.2 ± 5.8 °C, respectively, during the whole time required to complete the assays.

#### 3.1.1. Static System

No racking was performed during the three years of the experiment (minimum time to be considered Malt Whisky) in the static system, and it was periodically sampled over this interval to track the evolution at the following aging times: 0.5, 1, 2, 3, 4, 6, 8, 10, 12, 16, 20, 24, 28, 32, and 36 months. Two casks (*n* = 2) were used in order to duplicate the experiment.

#### 3.1.2. Dynamic System (Criadera and Solera)

**Figure 11:**
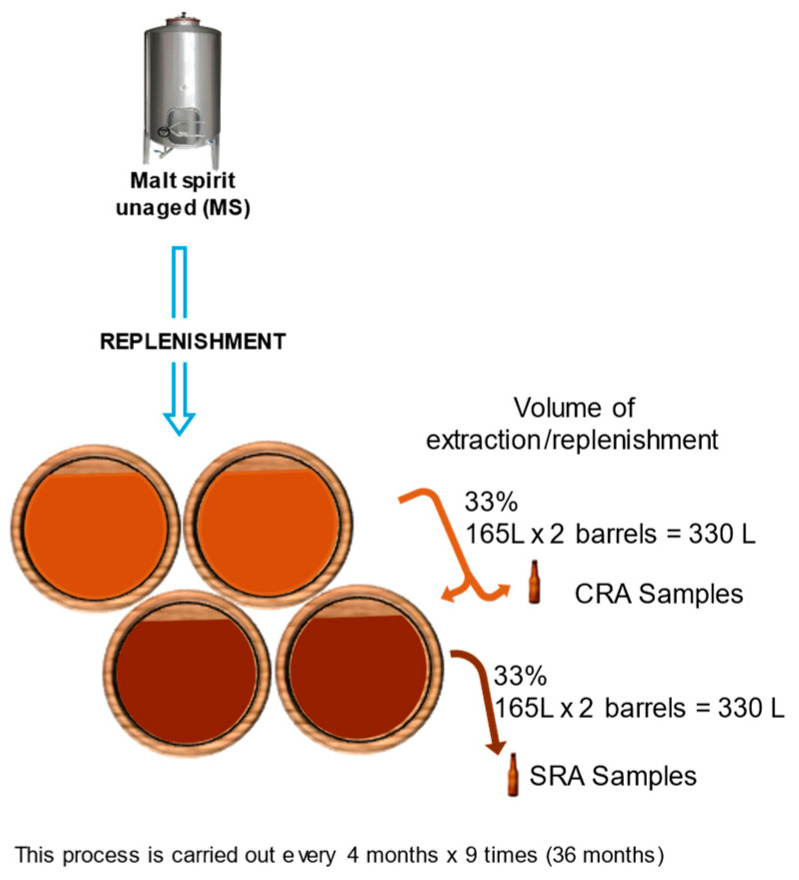
Extraction and replenishment operation.

A dynamic system consisting of a Criadera (CRA) and a Solera (SRA), both scales consisting of two barrels each, was set up. The dynamic system involves the partial emptying (1/3, 165 L) of the casks and their replenishment with distillate (either aged or unaged) depending on the scale of the system. This process is carried out every 4 months for three years. Sampling is carried out just before proceeding with the extraction and replenishment operations, and representative samples are taken from each of the two barrels that make up each scale in the system (Figure 11). In order to perform the experiment in duplicate, two sets of casks, each made of four casks, were used. After the 36 months of the study, a total of nine composite samples had been extracted, at 4, 8, 12, 16, 20, 24, 28, 32, and 36 months of aging, from each aging level and system. Table 5 shows the average age of the scales before the extraction and replenishment operations and calculated according to European normative [4].

The initial distillate was also analyzed. A total of 49 samples (15 static sampling × 2 casks + 9 dynamic samples × 1 scale (SRA) × 2 sets + 1 initial distillate) were analyzed in triplicate.

### 3.2. Oenological Control Parameters

All the oenological parameters were determined according to the official methods for the analysis of spirits described by the International Organization of Vine and Wine [26]. The alcoholic strength (% ABV) was obtained after the distillation of the sample by measuring the density of the distillate using a DMA-5000 density meter (Anton Paar, Ashland, OR, USA); the pH was determined by means of a Basic 20 pH meter (Crison Instruments SA, Barcelona, Spain) and the total acidity was determined by potentiometric titrations at pH 7.5 and was expressed as g acetic acid/L.

The volatile acids were determined using a segmented flow AA3 HR Autoanalyzer (Seal Analytical, Norderstedt Stadt, Germany), the results were expressed as g acetic acid/L.

### 3.3. Organic Acids

Ion chromatography was used for the analysis of organic acids using a 930 Compact IC Flex chromatograph (Metrohm, Madrid, Spain), equipped with a Metrosep Organic Acids column of 250 mm × 7.8 mm (i.d.) and 9 μm particle size. The method followed has been described in previous works [7]. Acetic, lactic, malic, succinic, and tartaric acids were identified. The data were processed and acquired using the software application MagicNet 3.3 (Metrohm, Madrid, Spain). The results were expressed as mg/L.

For the preparation of the eluents, ultrapure water (EMD Millipore, Bedford, MA, USA), 0.1 M sulfuric acid (Sigma-Aldrich, Saint Louis, MO, USA), and UHPLC quality acetone (VWR International, Radnor, PA, USA) were used. The tartaric acid for calibration was purchased from PanReac (Barcelona, Spain), and the rest of the standards from Sigma Aldrich (Saint Louis, MO, USA).

### 3.4. Aldehydes, Acetal, Methanol, Higher Alcohols, Esters, Glycerol and 2,3-Butanediol

An Agilent 7890B Gas Chromatograph (Agilent Technologies, Santa Clara, CA, USA) coupled to a flame ionization detector was used for the analysis of acetaldehyde, diethylacetal (1,1-diethoxyethane), methanol, higher alcohols, ethyl acetate, fatty acid esters, organic acid esters (except diethyl tartrate), glycerol and 2,3-butanediol. The methodology is described in previous work by our research group [5]. The samples were directly injected in triplicate. The results were expressed as mg/L.

Total acetaldehydes were obtained from the sum of the concentrations of acetaldehyde and its diethylacetal, expressing the diethylacetal as acetaldehyde (1 mg diethylacetal is equivalent to 0.373 mg acetaldehyde). The result was expressed as mg total acetaldehyde/L.

An Acquity UPLC chromatograph (Waters, Milford, MA, USA) equipped with a binary solvent system and an Acquity UPLC^®^ BEH C-18 column (2.1 × 50 mm^2^; 1.7 µm particle size) was used to determine diethyl tartrate. For mass spectroscopy determination, electrospray ionization (ESI) mass spectra were used with positive polarity and registered by a Xevo-G2S Q-TOF (Waters, Milford, MA, USA). The samples and standards were filtered through 0.22 µm nylon filters and injected in triplicate. The calibration line ranged from 0.001 mg/L to 0.5 mg/L. The compound was identified by comparing its retention time and mass spectra against those previously obtained for the standards. The results were expressed as mg/L.

The standards for the calibration of acetaldehyde, methanol, diethylacetal, higher alcohols, ethyl acetate, fatty acid esters, organic acid esters, glycerol, 2,3-butanediol, as well as the internal standards used, 2-pentanol and ethyl undecanoate, were purchased from Sigma Aldrich (Saint Louis, MO, USA). Ultrapure water (EMD Millipore, Bedford, MA, USA) and HPLC quality ethanol ≥ 99% (Scharlab, S.L., Barcelona, Spain) were used for the preparation of the standards.

### 3.5. Total Polyphenol Index

The Total Polyphenol Index (TPI) was determined by absorbance at 280 nm using a Lambda25 spectrophotometer (Perkin Elmer, Boston, MA, USA) and quartz cuvettes of 10 mm light path. For the quantification of the samples, a calibration curve was created with solutions of gallic acid at concentrations ranging from 0 to 50 mg/L. The samples were diluted at a 1:10 ratio in ultrapure water. The results were expressed as mg gallic acid equivalents (GAE)/L.

### 3.6. Phenolic Compounds and Furanic Aldehydes

The phenolic compounds and the furanic aldehydes were quantified by UHPLC according to the methodology developed by our research group [27,28]. A Waters Acquity UPLC unit equipped with a PDA detector and an Acquity UPLC C18 BEH column, 100 × 2.1 mm (i.d.) of 1.7 µm particle size (Waters Corporation, Milford, MA, USA) were employed. Seven phenolic acids: gallic acid, ellagic acid, vanillic acid, protocatechuic acid, caffeic acid, p-coumaric acid, syringic acid—5 phenolic aldehydes: p-hydroxybenzaldehyde, vanillin, syringaldehyde, coniferaldehyde, sinapaldehyde—and 3 furanic aldehydes: furfural, 5-methylfurfural, 5-hydroxymethylfurfural—were identified.

The samples and standards were filtered through nylon membranes with a pore size of 0.22 µm and injected in triplicate. The compounds were identified by comparison of the samples’ retention time and UV-Vis spectra with the standards. The calibration curves obtained covered the range from 0.1 mg/L to 20.0 mg/L. The results were expressed as mg/L.

To prepare the eluents for the determination of the phenolic compounds and furanic aldehydes, UHPLC-grade acetonitrile and acetic acid (PanReac, Barcelona, Spain), as well as ultrapure water (EMD Millipore, Bedford, MA, USA) and HPLC-grade ethanol ≥ 99% (Scharlab, S.L., Barcelona, Spain) were used. The standards for the calibrations were purchased from Sigma Aldrich (Saint Louis, MO, USA).

### 3.7. Sensory Analyses

The sensory evaluation sessions were conducted in a room at 22 °C. Thirty mL of each sample were poured into standard black wine glasses [29], which were covered with a watch glass until the time of evaluation to favor the concentration of the aromas. The two samples from each experience were combined at the same ratio in a single sample (*n* = 1), 72 h prior to tasting, the samples were diluted with demineralized water to 30% ABV. The panel consisted of 10 members, all of whom performed frequent sensory evaluations on this type of sample and were familiar with general tasting procedures. The panel was trained for the sensory evaluation of the samples. The first session focused on identifying and quantifying pre-selected descriptors in reference samples. These reference samples were available throughout five more sessions, during which panelists evaluated blind samples, including the references. The training was considered complete when all panelists accurately identified and quantified the descriptors in the reference samples.

The samples were analyzed by traditional Descriptive Quantitative Analysis (DQA) [30], using 5-point intensity scales (1: absent; 2: weak; 3: medium; 4: high; 5: very high). The descriptors were selected based on previous works carried out by our research team on brandies [5,31], and were supplemented with more specific and suitable descriptors for malt spirits [22]. Thus, the following were analyzed among the olfactory descriptors: pungent, malted cereal, boiled vegetables, vinous, nuts, spicy, toasted oak, aromatic intensity, soapy, and off-odor; and among the olfactory-gustatory descriptors: alcohol, smoothness, fatty, dryness, bitterness, balance and off-flavor.

Additionally, the judges were asked to place a number of samples from the dynamic Solera system extracted at 12, 24, and 36 months (SRA-12, SRA-24, and SRA-36) of aging time and according to an integral evaluation of each sample, on a scale based on static aging samples extracted at 4, 8, 12, 18, 24 and 36 months. The solera sample’s position on that scale was based on finding the sample from the static system that most closely resembled it.

### 3.8. Statistics

The physicochemical variables were subjected to Analysis of Variance (ANOVA) using Statgraphics 19 Software package (Statgraphics Technologies, Inc., The Plains, VA, USA), while Microsoft Excel 365 (Microsoft Corp., 141 Redmond, WA, USA) was employed to generate the graphics.

A statistical analysis of physicochemical variables was carried out in order to gain some insight into the correlation between the variables determined in the study and the dependent variable (average aging) in the statically aged malt spirits. This analysis was performed using the Statgraphics 19 Software package (Statgraphics Technologies, Inc., The Plains, VA, USA) and included the following steps: (1) Correlation analysis between all the study variables and the dependent variable (average age) in order to identify the linear relationships between them. (2) Principal factor analysis in order to explore the relationships between the study variables. (3) Two techniques were used to construct a predictive model for the average age as a function of the selected study variables. The model was trained using 75% of the static aging samples and validated with the remaining 25%. PLS (Partial Least Squares) and MLR (multiple linear regression) models were generated. These models allowed us to identify the variables with the most significant influence on the average age and to predict the average age of the samples extracted from the dynamic test.

The sensory analyses were subjected to Analysis of Variance (ANOVA) using Statistica 7.0 (StatSoft. Inc., Tulsa, OK, USA), while Microsoft Excel 2016 (Microsoft Corp., 141 Redmond, WA, USA) was employed to generate the spider charts.

## 4. Conclusions

The evolution of the same malt spirit aged using static and dynamic systems for 36 months has been studied, comparing the behavior of the compounds present in the distillate and those acquired from the Sherry Casks^®^ during aging.

Over the 36 months of aging in the static system, there was an increase in the concentration of compounds such as organic acids, ethyl esters, polyols (glycerol and 2,3-butanediol), phenolic compounds, and furanic aldehydes. The dynamic system was strongly influenced by the extraction and replenishment operations, with an initial increase that reached a maximum at 12–16 months of aging. The statistical analysis confirmed the trend observed in the static system, with a positive correlation with aging time greater than 0.96 for many of its variables, such as acetic acid, diethyl tartrate, diethyl succinate, and TPI, among others. In the FA, both Factors 1 and 2 explained 89.5% of the variability and allowed the classification of the aged spirits by aging time.

The multivariate regression model developed for the static system showed a strong correlation between the selected variables and the aging of the whisky or aged malt spirit, exhibiting a highly accurate prediction capacity. Thus, the selected MLR and PLS models predicted the aging of the samples quite closely to their actual age, with errors lower than 1.1 months for all the validated samples.

When the MLR and PLS models were used to estimate the aging of the spirits in the Solera of the dynamic system, the age predicted for the spirits in the first extractions was higher than the actual average age; however, as more extraction and replenishment operations had been completed, the difference between the two values decreased. Therefore, in the initial samples of the dynamic aging system, aged malt spirits are obtained with physicochemical characteristics of a malt spirit of greater age than the actual average age, but this deviation gradually disappears as the aging system stabilizes.

The effect of aging the malt spirit in Sherry Casks^®^ was positively perceived during the sensory evaluations, both for the static and the dynamic system. Certain attributes, such as malted cereal or boiled vegetables decreased, while toasted oak, vinous, or aromatic intensity increased. When both aging systems were compared in terms of average aging, there were no significant differences between the oldest spirits.

The results obtained over the three years of this study at an industrial scale provided valuable scientific information on the aging of spirits in Sherry Casks^®^ using either static or dynamic systems. Furthermore, the application of regression models has proven to be a useful tool for the industry to determine the variables and parameters that would allow them to control the aging processes in a more efficient and accurate manner.

## Figures and Tables

**Table 1:** Correlation coefficients of the variables studied with the aging time for static aging.

Variable	Correlation Coefficient	Variable	Correlation Coefficient
pH	−0.5033	2-phenyl ethanol	0.6987
Total acidity	0.9637	2.3-butanediol	0.8953
Volatile acids	0.9338	Benzaldehyde	0.6171
Total Phenolic Index	0.9766	Hexanol	0.3763
Acetaldehyde	0.5879	Diethyl malate	0.9716
Diethylacetal	0.9427	Gallic acid	0.9631
Total acetaldehydes	0.8308	5-hydroxymethylfurfural	0.9301
Methanol	0.6805	Protocatechuic acid	0.8349
N-propanol	0.2486	Furfural	0.8766
Ethyl acetate	0.9647	vanillic acid	0.8668
I-butanol	0.4943	p-hydroxybenzaldehyde	0.7410
N-butanol	−0.0397	5-methylfurfural	0.7786
2-methyl-1-butanol	0.3028	Syringic acid	0.9619
3-methyl-1-butanol	0.573	Vanillin	0.9555
Ethyl hexanoate	0.0902	Caffeic acid	0.9626
Ethyl octanoate	0.5401	p-coumaric acid	0.9568
Ethyl decanoate	−0.0258	Syringaldehyde	0.9567
Ethyl dodecanoate	0.8240	Coniferaldehyde	0.9680
Ethyl tetradecanoate	0.7889	Sinapaldehyde	0.9401
Ethyl hexadecanoate	0.9030	Ellagic acid	0.9374
Ethyl octadecanoate	0.9373	Tartaric acid	0.9182
Glycerol	0.9331	Malic acid	0.9191
Ethyl lactate	0.9257	Succinic acid	0.9184
Diethyl succinate	0.9779	Lactic acid	0.9237
Diethyl tartrate	0.9629	Acetic acid	0.9774

**Table 2:** Correlation coefficient of Factors 1 and 2 of the variables that present a high correlation (r > |0.40|) with aging time.

Variables	Factor 1	Factor 2	Variables	Factor 1	Factor 2
Total acidity	0.883	0.460	5-hydroxymethylfurfural	0.658	0.680
Volatile acids	0.843	0.442	Protocatechuic acid	0.778	0.431
Total Phenolic Index (TPI)	0.863	0.495	Furfural	0.620	0.653
Diethylacetal	0.824	0.500	Vanillic acid	0.767	0.501
Total acetaldehyde	0.847	-	p-hydroxybenzaldehyde	0.565	0.495
Methanol	0.607	-	5-methylfurfural	0.810	-
Ethyl acetate	0.764	0.584	Syringic acid	0.693	0.670
Ethyl dodecanoate	0.410	0.851	Vanillin	0.824	0.510
Ethyl tetradecanoate	-	0.915	Caffeic acid	0.772	0.579
Ethyl hexadecanoate	0.515	0.837	p-coumaric acid	0.751	0.612
Ethyl octadecanoate	0.599	0.743	Syringaldehyde	0.729	0.628
Glycerol	0.873	0.456	Coniferaldehyde	0.753	0.591
Ethyl lactate	0.902	-	Sinapaldehyde	0.783	0.487
Diethyl succinate	0.839	0.526	Ellagic acid	0.773	0.553
Diethyl tartrate	0.860	0.495	Tartaric acid	0.878	0.409
2-phenyl ethanol	0.591	0.506	Malic acid	0.906	-
2,3-butanediol	0.919	-	Succinic acid	0.918	-
Benzaldehyde	-	0.820	Lactic acid	0.904	-
Diethyl malate	0.736	0.618	Acetic acid	0.852	0.504
Gallic acid	0.753	0.570			

**Table 3:** Multiple Linear Regression Model used to determine the aging time of aged malt spirit.

Regression	R^2^ (Adjusted to DF)	Model *p*-Value (95%)	MAE
Average age = −10.4405 + 44.1798 × Total acidity + 3.86367 × Ethyl octadecanoate + 2.20228 × Diethyl succinate − 0.564349 × 2,3-butanediol − 2.13775 × Protocatechuic acid − 3.34393 × p-hydroxybenzaldehyde + 13.7756 × 5-methylfurfural + 6.80837 × Caffeic acid + 2.65815 × Sinapaldehyde	99.77	0.0000	0.41

MAE: Mean Absolute Error.

**Table 4:** Age, in months, of the malt spirits from the dynamic system as predicted by MLR and PLS regression models.

			MLR		PLS	
ID	Aging Time	Actual Average Age	Predicted Age	Difference with Actual Age *	Predicted Age	Difference with Actual Age *
SRA-8	8	8	18.6 ± 1.9	−10.6	17.6 ± 0.5	−9.6
SRA-12	12	11.6	27.1 ± 1.2	−15.5	24.7 ± 1.1	−13.1
SRA-16	16	14.5	29.1 ± 2.1	−14.6	27.4 ± 1.0	−12.9
SRA-20	20	16.9	27.9 ± 1.4	−11.0	26.3 ± 0.8	−9.4
SRA-24	24	18.7	28.9 ± 1.8	−10.2	27.0 ± 1.5	−8.3
SRA-28	28	20.1	27.3 ± 1.2	−7.2	25.8 ± 1.2	−5.7
SRA-32	32	21.2	26.3 ± 2.0	−5.1	24.7 ± 1.7	−3.5
SRA-36	36	22	24.1 ± 2.1	−2.1	22.3 ± 1.2	−0.3

* Difference with actual age (Predicted age − actual average age).

**Table 5:** Average age of the Criadera (CRA) and the Solera (SRA).

		Months									
Aging time		0	4	8	12	16	20	24	28	32	36
Average age	SRA	0	4.0	8.0	11.6	14.5	16.9	18.7	20.1	21.2	22.0
	CRA	0	4.0	6.7	8.4	9.6	10.4	10.9	11.3	11.5	11.7

## Data Availability

Data are contained within the article and Supplementary Materials.

## Supplementary Materials

The following supporting information can be downloaded at: https://www.mdpi.com/article/10.3390/molecules30061378/s1, Table S1: pH values for static ageing samples and dynamic ageing—Solera samples (SRA); Table S2: Acetal-dehyde, diethylacetal, total acetaldehydes, benzaldehyde and methanol (mg/L) for malt spirits in static ageing malt spirits and in the Solera (SRA) from the dynamic ageing system; Table S3: Higher alcohols (mg/L) for malt spirits in static ageing; Table S4: Higher alcohols (mg/L) for malt spirits in the Solera (SRA) from the dynamic ageing system; Table S5: Fatty acids ethyl esters (FAEE) (mg/L) for malt spirits in static ageing; Table S6: Fatty acid ethyl esters (FAEE) (mg/L) for malt spirits in the Solera (SRA) from the dynamic ageing system; Table S7: Predicted age and residuals of samples used to obtain a model by Multiple Linear Regression (MLR) and Partial Least Square (PLS); Table S8: Predicted age and residuals of 14 cases used to test the model obtained by Multiple Linear Regression (MLR); Table S9: Predicted age and residuals of 14 cases used to test the model obtained by Partial Least Square (PLS); Table S10: Contributions from the individual variables to the principal component in the regression model obtained through PLS; Table S11: Scores on the olfactory sensory descriptors of the samples and analysis of variance of the data for comparisons between samples according to average age and processing time; Table S12: Scores on the olfactory-gustatory sensory descriptors of the samples and analysis of variance of the data for comparisons between samples according to average; Figure S1: Evolution of (A) protocatechuic acid, (B) vanillic acid, (C) syringic acid, (D) caffeic acid, (E) p-hydroxybenzaldehyde, (F) vanillin, (G) 5-hydroxymethylfurfural (5-HMF) and (H) 5-methylfurfural for malt spirits in static ageing malt spirits and in the Solera (SRA) from the dynamic ageing system.
