# Supplementary material for: Chemical Characterization and Sensory Evaluation of Scottish Malt Spirit Aged in Sherry Casks®: Comparison Between Static and Dynamic Aging Systems
Source: Molecules. 2025 Mar 19;30(6):1378. doi: 10.3390/molecules30061378 (PMC11944592; doi:10.3390/molecules30061378)
Supplement: Supplementary file 1 [file molecules-30-01378-s001.zip › molecules-3469221-supplementary.pdf]

Supplementary Materials

Chemical Characterization and Sensory Evaluation of Scottish Malt Spirit Aged in Sherry Casks®: Comparison Between Static and Dynamic Aging Systems

Daniel Butrón-Benítez [1,2], Manuel J. Valcárcel-Muñoz [3], M. Valme García-Moreno [1,*], M. Carmen Rodríguez-Dodero [1] and Dominico A. Guillén-Sánchez [1,*]

1 Departamento de Química Analítica, Facultad de Ciencias, Instituto Investigación Vitivinícola y Agroalimentaria (IVAGRO), Universidad de Cádiz, Campus Universitario de Puerto Real, 11510 Puerto Real, Cádiz, Spain. D.B.-B.: daniel.butron@uca.es; M.C.R.-D.: maricarmen.dodero@uca.es

2 Bodegas Fundador S.L.U., C/ San Ildefonso, nº 3, 11403 Jerez de la Frontera, Cádiz, Spain.

3 Oeno R&D FL, 11402 Jerez de la Frontera, Cádiz, Spain. M.J.V.-M.: mjc.valcarcel@gmail.com

* Correspondence: M.V.G.-M.: valme.garcia@uca.es; D.A.G.-S.: dominico.guillen@uca.es Tel.: +34-956016456

Table S1. pH values for static ageing samples and dynamic ageing – Solera samples (SRA).

Time process (months)	pH	
	Static ageing	Dynamic ageing (SRA)
0	5.21 ± 0.00 [f]	5.21 ± 0.00 [e]
0.5	4.57 ± 0.01 [e]	
1	4.54 ± 0.01 [d, e]	
2	4.54 ± 0.00 [d, e]	
3	4.49 ± 0.01 [b, c, d, e]	
4	4.48 ± 0.02 [b,c,d,e]	4.24 ± 0.01 [a]
6	4.46 ± 0.01 [a, b, c, d]	
8	4.48 ± 0.02 [b, c, d, e]	4.33 ± 0.01 [b]
10	4.48 ± 0.02 [b, c, d, e]	
12	4.50 ± 0.01 [c, d, e]	4.35 ± 0.01 [c]
16	4.47 ± 0.02 [a, b, c, d]	4.32 ± 0.01 [b]
20	4.38 ± 0.06 [a]	4.32 ± 0.01 [b]
24	4.43 ± 0.03 [a, b, c]	4.37 ± 0.00 [d]
28	4.38 ± 0.02 [a]	4.35 ± 0.01 [c]
32	4.40 ± 0.08 [a, b]	4.37 ± 0.01 [c, d]
36	4.41 ± 0.08 [a, b, c]	4.38 ± 0.00 [d]

Mean ± standard deviation (n=6); The significant differences in a particular column between the values obtained from the Tukey HSD test ($p < 0.05$) are indicated by a different letter.

Table S2. Acetaldehyde, diethylacetal, total acetaldehydes, benzaldehyde and methanol (mg/L) for malt spirits in static ageing malt spirits and in the Solera (SRA) from the dynamic ageing system.

Time process (months)	Acetaldehyde		Diethylacetal		Total acetaldehydes		Benzaldehyde		Methanol	
	Static ageing	SRA	Static ageing	SRA	Static ageing	SRA	Static ageing	SRA	Static ageing	SRA
0	55.8 ± 0.7	55.8 ± 0.7	76.1 ± 0.3	76.1 ± 0.3	84.2 ± 0.6 ^{a}	84.2 ± 0.6 $^{a, b}$	0.5 ± 0.1 ^{e}	0.5 ± 0.1 $^{b, c}$	47.9 ± 0.3 $^{a, b, c}$	47.9 ± 0.3 $^{a, b}$
0.5	60.2 ± 3.5		77.2 ± 4.7		89.0 ± 5.2 $^{a, b, c}$		0.3 ± 0.0 ^{a}		48.3 ± 3.0 $^{a, b}$	
1	58.8 ± 1.0		75.9 ± 0.4		87.1 ± 1.1 $^{a, b}$		0.3 ± 0.0 ^{a}		46.9 ± 0.6 ^{a}	
2	60.2 ± 0.8		76.8 ± 0.6		88.9 ± 0.7 $^{a, b, c}$		0.3 ± 0.0 ^{a}		47.1 ± 0.5 $^{a, b}$	
3	61.5 ± 1.4		78.5 ± 0.6		90.8 ± 1.3 $^{a, b, c, d}$		0.3 ± 0.0 $^{a, b}$		47.8 ± 0.7 $^{a, b}$	
4	61.1 ± 1.0	60.5 ± 2.7	77.3 ± 2.1	83.7 ± 1.6	90.0 ± 1.6 $^{a, b, c, d}$	91.7 ± 3.1 $^{c, d}$	0.4 ± 0.0 $^{a, b, c}$	0.4 ± 0.0 $^{a, b}$	48.0 ± 0.3 $^{a, b}$	48.6 ± 0.7 $^{a, b, c}$
6	62.6 ± 1.3		82.3 ± 3.1		93.3 ± 2.4 $^{b, c, d, e}$		0.3 ± 0.0 $^{a, b}$		49.0 ± 0.8 $^{a, b, c}$	
8	63.8 ± 0.5	60.7 ± 0.9	82.4 ± 2.4	84.9 ± 0.4	94.5 ± 1.1 $^{c, d, e}$	92.4 ± 0.9 $^{c, d, e}$	0.3 ± 0.0 ^{a}	0.4 ± 0.0 $^{b, c}$	48.6 ± 0.5 $^{a, b, c}$	47.9 ± 0.2 ^{a}
10	62.7 ± 0.4		84.7 ± 1.3		94.3 ± 0.8 $^{c, d, e}$		0.4 ± 0.0 $^{a, b, c, d}$		50.1 ± 0.6 $^{a, b, c}$	
12	65.1 ± 1.9	63.6 ± 0.7	86.8 ± 2.9	91.4 ± 1.6	97.5 ± 2.8 $^{e, f}$	97.7 ± 1.2 ^{e}	0.4 ± 0.0 $^{a, b, c, d}$	0.5 ± 0.0 ^{c}	50.7 ± 0.8 $^{a, b, c}$	49.9 ± 1.0 ^{c}
16	62.3 ± 2.5	61.0 ± 0.8	90.6 ± 1.4	90.7 ± 0.8	96.1 ± 2.5 $^{d, e, f}$	94.8 ± 1.0 $^{d, e}$	0.4 ± 0.0 $^{a, b, c, d}$	0.5 ± 0.0 ^{c}	50.1 ± 2.5 $^{a, b, c}$	49.7 ± 0.6 $^{b, c}$
20	64.3 ± 4.5	57.3 ± 1.0	89.6 ± 1.7	86.2 ± 0.4	97.7 ± 4.7 $^{e, f}$	89.4 ± 1.0 $^{b, c}$	0.4 ± 0.0 $^{b, c, d, e}$	0.5 ± 0.1 ^{c}	52.4 ± 5.1 $^{a, b, c}$	47.7 ± 0.5 ^{a}
24	65.8 ± 1.7	58.4 ± 1.3	95.8 ± 1.7	88.6 ± 0.5	101.5 ± 1.9 ^{f}	91.5 ± 1.4 $^{c, d}$	0.4 ± 0.0 $^{a, b, c, d}$	0.4 ± 0.0 $^{b, c}$	54.9 ± 4.8 ^{c}	48.4 ± 0.4 $^{a, b, c}$
28	62.8 ± 2.6	56.7 ± 2.2	93.2 ± 1.5	86.3 ± 1.5	97.5 ± 3.1 $^{e, f}$	88.8 ± 2.7 $^{b, c}$	0.4 ± 0.0 $^{b, c, d, e}$	0.4 ± 0.0 $^{b, c}$	53.4 ± 3.8 $^{b, c}$	48.8 ± 0.9 $^{a, b, c}$
32	65.0 ± 2.1	55.1 ± 0.5	96.7 ± 1.5	82.2 ± 1.2	101.1 ± 2.4 ^{f}	85.8 ± 0.9 $^{a, b}$	0.4 ± 0.0 $^{c, d, e}$	0.4 ± 0.0 $^{a, b}$	53.0 ± 3.1 $^{a, b, c}$	48.2 ± 0.7 $^{a, b}$
36	64.9 ± 1.5	53.5 ± 3.2	97.2 ± 1.7	78.2 ± 3.9	101.2 ± 1.9 ^{f}	82.7 ± 4.5 ^{a}	0.4 ± 0.0 $^{d, e}$	0.3 ± 0.0 ^{a}	52.6 ± 1.4 $^{a, b, c}$	48.5 ± 0.6 $^{a, b, c}$

Mean ± standard deviation (n=6); The significant differences in a particular column between the values obtained from the Tukey HSD test ($p< 0.05$) are indicated by a different letter.

Table S3. Higher alcohols (mg/L) for malt spirits in static ageing.

Time process (months)	N-propanol	I-butanol	N-butanol	2-phenyl ethanol	Hexanol	2-methyl-1-butanol	3-methyl-1-butanol	Total higher alcohols
0	336.1 ± 1.3	561.1 ± 0.0	11.7 ± 0.0	45.2 ± 0.3	1.5 ± 0.0	414.8 ± 1.3	1093.6 ± 0.2	2464.0 ± 2.1 [a]
0.5	321.7 ± 3.6	534.9 ± 2.9	11.5 ± 0.4	43.9 ± 0.4	1.4 ± 0.1	398.6 ± 2.3	1054.5 ± 6.2	2366.7 ± 10.5 [d]
1	319.1 ± 1.9	540.2 ± 2.6	11.7 ± 0.2	44.4 ± 0.4	1.4 ± 0.1	399.1 ± 4.6	1054.0 ± 11.3	2369.9 ± 20.4 [d]
2	317.8 ± 1.4	538.5 ± 1.5	11.5 ± 0.3	44.7 ± 0.4	1.4 ± 0.0	399.3 ± 1.6	1056.8 ± 3.3	2369.9 ± 6.9 [d]
3	322.5 ± 1.7	543.2 ± 2.0	11.5 ± 0.8	45.6 ± 1.4	1.5 ± 0.1	402.8 ± 2.0	1066.4 ± 8.4	2393.5 ± 10.7 [c, d, e]
4	318.3 ± 0.6	536.0 ± 1.0	11.5 ± 0.4	45.4 ± 0.6	1.4 ± 0.1	397.5 ± 1.0	1051.8 ± 3.7	2361.8 ± 3.7 [d]
6	321.9 ± 2.4	540.2 ± 2.8	11.3 ± 0.5	45.2 ± 0.3	1.4 ± 0.1	398.3 ± 1.4	1052.5 ± 3.2	2370.9 ± 6.4 [d]
8	317.8 ± 2.5	536.7 ± 2.6	11.3 ± 0.3	45.8 ± 0.4	1.5 ± 0.0	396.8 ± 1.1	1050.7 ± 2.3	2360.6 ± 4.4 [d]
10	317.7 ± 1.3	538.1 ± 1.9	11.1 ± 0.4	46.3 ± 0.6	1.5 ± 0.1	396.5 ± 2.9	1050.3 ± 7.1	2361.6 ± 12.2 [d]
12	318.0 ± 5.1	536.8 ± 7.3	11.1 ± 0.2	46.1 ± 0.6	1.5 ± 0.0	396.5 ± 4.6	1052.0 ± 14.2	2361.9 ± 31.0 [d]
16	321.0 ± 2.3	542.4 ± 2.8	11.2 ± 0.5	47.4 ± 0.3	1.5 ± 0.0	402.1 ± 5.9	1064.0 ± 3.5	2389.6 ± 11.9 [c, d, e]
20	319.9 ± 2.1	540.2 ± 2.9	11.0 ± 0.8	47.2 ± 0.7	1.5 ± 0.0	399.9 ± 5.3	1059.4 ± 4.3	2379.1 ± 13.9 [d, e]
24	326.2 ± 2.6	551.0 ± 5.2	11.1 ± 0.5	45.6 ± 0.2	1.5 ± 0.0	408.4 ± 6.1	1081.1 ± 8.6	2424.9 ± 21.2 [a,b,c]
28	319.6 ± 2.4	541.0 ± 2.2	11.0 ± 0.9	46.9 ± 0.5	1.5 ± 0.1	399.0 ± 2.4	1064.6 ± 2.3	2383.5 ± 7.3 [d, e]
32	325.0 ± 3.6	549.1 ± 5.6	11.4 ± 0.5	46.9 ± 0.5	1.5 ± 0.1	404.1 ± 4.3	1077.4 ± 11.2	2415.4 ± 24.1 [b, c, d]
36	326.0 ± 3.6	556.1 ± 5.4	12.1 ± 0.5	47.0 ± 0.5	1.5 ± 0.2	408.6 ± 3.7	1092.4 ± 5.6	2443.6 ± 17.3 [a, b]

Mean ± standard deviation (n=6); The significant differences in a particular column between the values obtained from the Tukey HSD test ($p < 0.05$) are indicated by a different letter.

Table S4. Higher alcohols (mg/L) for malt spirits in the Solera (SRA) from the dynamic ageing system.

Time process (months)	N-propanol	I-butanol	N-butanol	2-phenyl ethanol	Hexanol	2-methyl-1-butanol	3-methyl-1-butanol	Total higher alcohols
0	336.1 ± 1.3	561.1 ± 0.0	11.7 ± 0.0	45.2 ± 0.3	1.5 ± 0.0	414.8 ± 1.3	1093.6 ± 0.2	2464.0 ± 2.1 [a]
4	318.4 ± 6.9	540.5 ± 2.4	10.5 ± 0.5	45.2 ± 1.1	1.7 ± 0.0	401.8 ± 3.0	1046.1 ± 4.3	2364.2 ± 16.1 [b]
8	311.7 ± 1.9	523.4 ± 4.1	10.1 ± 0.3	47.5 ± 1.1	1.6 ± 0.1	392.7 ± 4.8	1014.1 ± 10.0	2301.1 ± 19.6 [c]
12	317.4 ± 2.9	532.4 ± 3.6	10.1 ± 0.3	48.3 ± 0.4	1.7 ± 0.1	398.5 ± 1.7	1030.1 ± 6.0	2338.5 ± 13.6 [b, c]
16	321.2 ± 3.8	540.5 ± 5.2	10.4 ± 0.5	48.9 ± 0.9	1.6 ± 0.0	404.0 ± 2.9	1044.9 ± 12.1	2371.5 ± 23.5 [c]
20	317.0 ± 2.3	532.1 ± 3.4	10.3 ± 0.4	48.8 ± 1.5	1.6 ± 0.1	402.8 ± 3.7	1030.3 ± 7.2	2343.0 ± 15.0 [b, c]
24	320.6 ± 1.1	536.9 ± 1.5	10.2 ± 0.3	49.3 ± 1.1	1.6 ± 0.0	402.8 ± 1.5	1035.3 ± 3.9	2356.9 ± 6.8 [b]
28	323.1 ± 4.5	543.3 ± 6.5	10.3 ± 0.1	49.4 ± 0.4	1.7 ± 0.0	406.0 ± 4.2	1045.3 ± 12.2	2379.1 ± 26.9 [b]
32	321.9 ± 1.5	539.7 ± 4.1	10.4 ± 0.4	48.3 ± 0.8	1.6 ± 0.1	402.8 ± 2.2	1037.3 ± 7.5	2362.1 ± 15.0 [b]
36	325.6 ± 2.2	544.5 ± 5.6	10.6 ± 0.8	47.9 ± 1.0	1.6 ± 0.1	399.6 ± 8.3	1044.1 ± 17.1	2373.9 ± 21.0 [b]

Mean ± standard deviation (n=6); The significant differences in a particular column between the values obtained from the Tukey HSD test ($p < 0.05$) are indicated by a different letter.

Table S5. Fatty acids ethyl esters (FAEE) (mg/L) for malt spirits in static ageing.

Time process (months)	Ethyl hexanoate	Ethyl octanoate	Ethyl decanoate	Ethyl dodecanoate	Ethyl tetradecanoate	Ethyl hexadecanoate	Ethyl octadecanoate	Total FAEE
0	2.9 ± 0.1	15.5 ± 0.1	53.1 ± 0.0	45.4 ± 0.0	10.2 ± 0.2	37.3 ± 0.4	1.2 ± 0.0	165.6 ± 0.6 [d, e, f]
0.5	2.8 ± 0.0	16.2 ± 0.1	51.3 ± 0.3	43.1 ± 0.3	9.7 ± 0.1	35.0 ± 0.2	1.1 ± 0.0	159.2 ± 0.9 [a]
1	2.9 ± 0.0	16.6 ± 0.2	51.6 ± 0.2	43.3 ± 0.1	9.7 ± 0.1	35.4 ± 0.2	1.2 ± 0.1	160.6 ± 0.7 [a, b]
2	2.9 ± 0.0	16.9 ± 0.1	51.8 ± 0.1	43.3 ± 0.2	9.7 ± 0.1	35.4 ± 0.4	1.1 ± 0.0	161.3 ± 0.7 [a, b, c]
3	3.0 ± 0.1	17.6 ± 0.6	53.2 ± 1.3	43.7 ± 0.5	10.0 ± 0.3	36.5 ± 0.9	1.2 ± 0.1	165.1 ± 3.4 [d, e]
4	3.0 ± 0.1	17.4 ± 0.3	52.8 ± 0.3	43.8 ± 0.3	9.8 ± 0.1	35.6 ± 0.1	1.1 ± 0.1	163.5 ± 1.2 [b, c, d]
6	3.1 ± 0.0	17.7 ± 0.1	52.8 ± 0.3	43.9 ± 0.2	9.8 ± 0.1	35.6 ± 0.1	1.2 ± 0.0	164.1 ± 0.7 [c, d, e]
8	3.1 ± 0.0	17.6 ± 0.1	52.6 ± 0.1	44.1 ± 0.4	9.9 ± 0.2	35.9 ± 0.1	1.2 ± 0.0	164.3 ± 0.9 [c, d, e]
10	3.2 ± 0.1	17.5 ± 0.1	52.6 ± 0.1	43.9 ± 0.1	9.7 ± 0.0	36.3 ± 0.3	1.2 ± 0.1	164.5 ± 0.3 [d, e]
12	3.2 ± 0.0	17.6 ± 0.1	52.2 ± 0.1	43.7 ± 0.1	9.6 ± 0.0	36.0 ± 0.2	1.1 ± 0.1	163.5 ± 0.4 [b, c, d]
16	2.8 ± 0.3	17.6 ± 0.2	53.0 ± 0.8	44.9 ± 0.1	10.0 ± 0.0	38.3 ± 0.6	2.0 ± 0.2	168.7 ± 1.8 [f, g, h]
20	2.7 ± 0.2	17.2 ± 0.2	52.7 ± 0.1	45.3 ± 0.1	10.4 ± 0.1	39.4 ± 0.5	2.4 ± 0.1	170.0 ± 0.9 [g, h]
24	2.7 ± 0.2	17.2 ± 0.2	52.2 ± 0.0	44.6 ± 0.1	10.1 ± 0.1	38.0 ± 0.7	2.2 ± 0.1	167.0 ± 1.0 [e, f, g]
28	2.8 ± 0.2	17.4 ± 0.1	52.5 ± 0.3	45.0 ± 0.2	10.3 ± 0.1	39.2 ± 0.7	2.6 ± 0.2	169.7 ± 1.0 [g, h]
32	3.1 ± 0.2	18.0 ± 0.4	52.0 ± 0.7	45.5 ± 0.1	10.4 ± 0.0	39.6 ± 0.2	2.6 ± 0.2	171.3 ± 0.1 [h, i]
36	3.3 ± 0.2	18.0 ± 0.3	52.0 ± 0.7	45.9 ± 0.1	10.6 ± 0.1	40.6 ± 0.2	2.8 ± 0.3	173.3 ± 0.3 [i]

Mean ± standard deviation (n=6); The significant differences in a particular column between the values obtained from the Tukey HSD test ($p < 0.05$) are indicated by a different letter.

Table S6. Fatty acid ethyl esters (FAEE) (mg/L) for malt spirits in the Solera (SRA) from the dynamic ageing system.

Time process (months)	Ethyl hexanoate	Ethyl octanoate	Ethyl decanoate	Ethyl dodecanoate	Ethyl tetradecanoate	Ethyl hexadecanoate	Ethyl octadecanoate	Total FAEE
0	2.9 ± 0.1	15.5 ± 0.1	53.1 ± 0.0	45.4 ± 0.0	10.2 ± 0.2	37.3 ± 0.4	1.2 ± 0.0	165.6 ± 0.6 [a]
4	3.1 ± 0.1	18.4 ± 0.5	56.7 ± 1.3	46.5 ± 1.2	10.4 ± 0.2	33.3 ± 1.8	1.4 ± 0.1	169.8 ± 1.9 [a]
8	3.1 ± 0.2	21.1 ± 0.3	55.5 ± 0.1	47.5 ± 0.1	10.5 ± 0.1	36.1 ± 0.7	2.3 ± 0.1	176.1 ± 1.0 [b]
12	3.3 ± 0.1	21.8 ± 0.3	55.8 ± 1.0	48.2 ± 0.0	10.8 ± 0.0	38.2 ± 0.2	2.7 ± 0.1	180.8 ± 1.6 [c, d]
16	3.2 ± 0.0	21.6 ± 0.2	56.1 ± 0.1	48.5 ± 0.2	10.8 ± 0.1	38.5 ± 0.7	2.8 ± 0.1	181.5 ± 1.3 [d]
20	3.1 ± 0.0	20.6 ± 0.3	54.7 ± 1.1	48.6 ± 0.1	11.0 ± 0.1	39.2 ± 0.4	2.9 ± 0.0	180.1 ± 0.6 [c, d]
24	3.0 ± 0.0	20.4 ± 0.3	55.9 ± 0.3	49.1 ± 0.3	11.1 ± 0.1	39.6 ± 0.3	2.9 ± 0.1	182.1 ± 1.1 [d]
28	2.9 ± 0.1	19.7 ± 0.1	55.7 ± 0.2	49.3 ± 0.1	11.1 ± 0.0	39.7 ± 0.2	2.9 ± 0.0	181.3 ± 0.5 [d]
32	2.8 ± 0.1	19.1 ± 0.2	55.1 ± 0.1	48.9 ± 0.4	11.1 ± 0.1	39.4 ± 0.9	2.9 ± 0.1	179.4 ± 1.4 [b, c, d]
36	2.8 ± 0.2	18.5 ± 0.5	54.6 ± 0.1	48.6 ± 0.6	11.1 ± 0.1	39.2 ± 1.8	2.8 ± 0.2	177.5 ± 2.6 [b, c]

Mean ± standard deviation (n=6); The significant differences in a particular column between the values obtained from the Tukey HSD test ($p < 0.05$) are indicated by a different letter

Table S7. Predicted age and residuals of samples used to obtain a model by Multiple Linear Regression (MLR) and Partial Least Square (PLS).

	MLR		PLS	
Time (months)	Predicted age (months)	Residuals	Predicted age (months)	Residuals
1	1.3	-0.3	1.0	0.2
2	2.2	-0.2	2.4	-0.8
3	3.2	-0.2	3.1	-0.4
4	4.0	0.0	4.3	-0.8
6	5.5	0.5	5.6	0.5
8	7.6	0.4	7.2	1.1
10	10.0	0.0	9.9	-0.1
12	12.4	-0.4	12.1	-0.3
16	17.1	-1.1	16.9	-0.5
20	20.3	-0.3	20.0	0.7
24	24.2	-0.2	24.7	-0.8
28	27.8	0.2	27.7	0.6
32	32.0	0.0	32.3	-0.3
36	35.9	0.1	35.9	0.4

Table S8. Predicted age and residuals of 14 cases used to test the model obtained by Multiple Linear Regression (MLR).

Time (months)	Predicted age (months)	Residuals
1	1.2	0.2
2	2.2	0.2
3	3.3	0.3
4	3.6	-0.4
6	5.9	-0.1
8	7.5	-0.5
10	10.4	0.4
12	12.5	0.5
16	17.6	1.6
20	21.2	1.2
24	24.1	0.1
28	28.2	0.2
32	32.1	0.1
36	35.7	-0.3

Table S9. Predicted age and residuals of 14 cases used to test the model obtained by Partial Least Square (PLS).

Time (months)	Predicted age (months)	Residuals
1	1.3	0.3
2	2.3	0.3
3	2.9	-0.1
4	3.9	-0.1
6	5.5	-0.5
8	6.6	-1.4
10	9.7	-0.3
12	12.0	0.0
16	16.8	0.8
20	21.1	1.1
24	25.4	1.4
28	27.8	-0.2
32	32.8	0.8
36	36.3	0.3

Table S10. Contributions from the individual variables to the principal component in the regression model obtained through PLS.

Variable	Coeficient		Coeficient
Ethyl acetate	0,021	Protocatechuic acid	-0,052
Total acidity	0,066	Furfural	-0,065
Volatile acids	0,065	vainillic acid	-0,022
Total Polyphenol indez (TPI)	0,077	p-Hydroxybenzaldehide	-0,015
Diethylacetal	0,013	5-Methylfurfural	0,011
Total acetaldehyde	-0,031	Syringic acid	0,073
Methanol	0,037	Vanillin	0,035
Ehtyl dodecanoate	0,029	Caffeic acid	0,052
Ehtyl hexadecanoate	0,043	p-coumaric acid	0,046
Ethyl octadecanoate	0,159	Syringaldehyde	-0,056
Glycerol	-0,032	Coniferylaldehyde	0,077
Ethyl Lactate	-0,025	Synapaldehyde	0,188
Diethyl Succinate	0,065	Ellagic acid	0,003
Diethyl Tartrate	0,055	Tartaric acid	0,012
2-phenyl ethanol	-0,027	Malic acid	0,002
2,3-butanodiol	-0,037	Succinic acid	-0,022
Diethyl malate	0,087	Lactic acid	0,080
Gallic acid	0,036	Acetic acid	0,069
5-Hydroxymethylfurfural	0,006		

Table S11. Scores on the **olfactory** sensory descriptors of the samples and analysis of variance of the data for comparisons between samples according to average age and processing time.

ID		Pungent	Malted cereal	Boiled vegetables	Vinous	Nuts	Toasted oak	Spicy	Aromatic intensity	Soapy
Static 4 months		2.3±0.6	3.7±0.6	2.7±1.5	2.0±0.0	1.3±0.6	1.7±0.6	1.7±0.6	2.0±0.0	1.7±1.1
Static 8 months		3.0±0.0	2.3±0.6	2.0±1.0	2.3±0.6	2.3±0.4	2.0±0.0	1.7±1.2	2.7±0.6	1.3±0.6
Static 12 months		2.3±0.6	1.7±1.2	1.3±0.6	3.0±0.0	2.3±0.6	2.3±0.6	2.3±0.6	2.7±0.7	1.3±0.3
Static 18 months		2.3±0.7	1.7±1.2	1.7±1.2	3.3±0.6	2.7±1.2	3.0±0.0	3.0±1.0	3.7±0.4	1.3±0.4
Static 24 months		3.3±0.6	1.7±1.2	1.3±0.6	3.7±0.6	3.3±0.6	3.3±0.6	3.0±1.0	3.7±1.2	1.0±0.0
Static 36 months		3.3±0.3	1.3±0.6	1.3±0.6	5.0±0.0	4.0±1.0	3.3±0.5	4.0±1.0	5.0±0.0	1.0±0.0
SRA-12		3.3±0.4	1.7±1.2	1.7±1.2	3.0±0.0	2.7±0.6	2.7±0.6	2.7±0.6	3.3±0.7	1.3±0.6
SRA-24		3.7±0.6	1.7±1.2	1.3±0.6	3.3±1.2	3.0±1.0	3.0±0.0	3.3±0.6	3.7±0.6	1.0±0.0
SRA-36		2.7±0.6	1.7±1.2	1.3±0.6	4.0±1.0	3.3±0.6	3.0±0.0	3.3±0.6	4.0±0.0	1.0±0.0
Analysis of variance by average age	*Average age*									
Static 12 m. vs SRA-12	12m	0.008*	-	0.358	-	0.153	0.153	0.153	0.071	-
Static 18 m. vs SRA-24	18m	0.047*	1.000	0.678	1.000	0.725	-	0.643	1.000	0.374
Static 24 m. vs SRA-36	24m	0.230	1.000	1.000	0.643	1.000	0.374	0.643	0.643	-
Analysis of variance by process time	*Time*									
Static 24 m. vs SRA-24	24m	0.519	1.000	1.000	0.678	0.643	0.374	0.643	1.000	-
Static 36 m. vs SRA-36	36m	0.012	0.320	-	0.005*	0.074	0.074	0.074	-	-

Mean ± standard deviation; ANOVA results are indicated by p-values (included in the second part of the table).
p-values less than 0.05 (marked with *) indicate significant differences between samples. Some p-values could not be calculated due to the absence of variation between the data of the samples compared.

Table S12. Scores on the **olfactory-gustatory** sensory descriptors of the samples and analysis of variance of the data for comparisons between samples according to average age and processing time.

ID		Alcohol	Smoothness	Fatty	Dryness	Bitterness	Balance
Static 4 months		2.7±1.5	2.0±1.0	1.7±0.6	2.3±1.2	1.7±0.3	1.7±0.6
Static 8 months		2.0±1.0	2.0±1.0	1.0±0.0	2.7±0.6	2.3±0.6	1.7±1.2
Static 12 months		2.7±0.6	2.3±0.6	1.3±0.6	2.3±0.6[a]	2.3±0.6	2.0±1.0
Static 18 months		2.7±0.6	1.7±1.2	1.0±0.0	3.0±1.0	2.0±0.0	2.3±1.5
Static 24 months		2.0±0.1	1.7±1.3	1.0±0.0	2.7±0.6	1.7±0.5	3.0±1.7
Static 36 months		2.0±0.0	2.0±1.0	1.0±0.0	2.3±0.6	1.7±0.4	4.7±0.6
SRA-12		2.3±0.4	1.7±1.2	1.3±0.6	2.0±0.0	2.3±0.6	2.0±1.0
SRA-24		2.3±0.6	2.3±0.5	1.0±0.0	2.0±0.0	1.3±0.6	3.0±1.0
SRA-36		2.7±0.6	2.0±1.0	1.0±0.0	3.0±1.0	2.3±0.6	3.0±1.0
Analysis of variance by average age	*Average age*						
Static 12 m. vs SRA-12	12m	0.096	0.174	-	0.131	-	-
Static 18 m. vs SRA-24	18m	0.519	0.422	-	0.158	0.116	0.561
Static 24 m. vs SRA-36	24m	0.116	0.725	-	0.643	0.230	1.000
Analysis of variance by process time	*Time*						
Static 24 m. vs SRA-24	24m	0.374	0.422	-	0.116	0.519	1.000
Static 36 m. vs SRA-36	36m	0.002*	-	-	0.074	0.017*	0.001*

Mean ± standard deviation; ANOVA results are indicated by p-values (included in the second part of the table).
p-values less than 0.05 (marked with *) indicate significant differences between samples. Some p-values could not be calculated due to the absence of variation between the data of the samples compared.

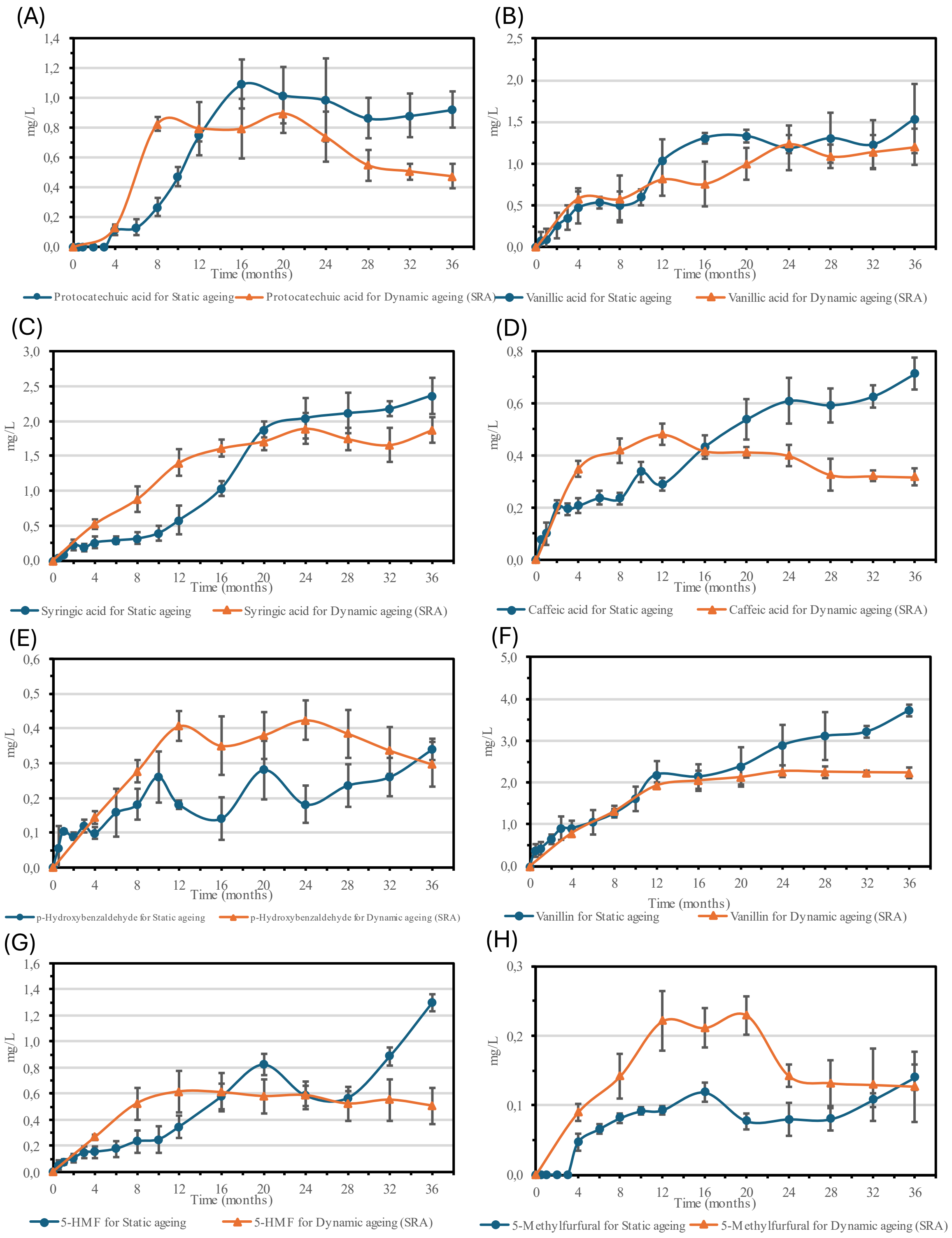

Figure S1. Evolution of (A) protocatechuic acid, (B) vanillic acid, (C) syringic acid, (D) caffeic acid, (E) p-hydroxybenzaldehyde, (F) vanillin, (G) 5-hydroxymethylfurfural (5-HMF) and (H) 5-methylfurfural for malt spirits in static ageing malt spirits and in the Solera (SRA) from the dynamic ageing system.
